# The Deployment of Routing Protocols in Distributed Control Plane of SDN

**DOI:** 10.1155/2014/918536

**Published:** 2014-08-28

**Authors:** Zhou Jingjing, Cheng Di, Wang Weiming, Jin Rong, Wu Xiaochun

**Affiliations:** College of Information & Electronic Engineering, Zhejiang Gongshang University, Hangzhou 310018, China

## Abstract

Software defined network (SDN) provides a programmable network through decoupling the data plane, control plane, and application plane from the original closed system, thus revolutionizing the existing network architecture to improve the performance and scalability. In this paper, we learned about the distributed characteristics of Kandoo architecture and, meanwhile, improved and optimized Kandoo's two levels of controllers based on ideological inspiration of RCP (routing control platform). Finally, we analyzed the deployment strategies of BGP and OSPF protocol in a distributed control plane of SDN. The simulation results show that our deployment strategies are superior to the traditional routing strategies.

## 1. Introduction

With the rapid development of science and technology, the old-fashioned network architectures can not meet the various needs for modern life. People want to change the existing network architecture urgently and set out to redesign new network architecture. The future network should have these properties, such as the underlying data plane which is dumb, simple, and minimal, the separation of control plane and data plane, the control plane which can completely control the entire network, and the upper layer which provides a common application programming interface of external parts. In this way, researchers can program via calling API (application programming interface) on the control plane to achieve innovation of network.

Software defined network is the powerful enabler of owing innovative network ideas. SDN revolutionizes the existing network architecture to provide the methods of programmable networks and decouples network architecture into the data plane, control plane, and control plane applications. So, SDN separates the data plane and control plane and, at the same time, improves the performance and scalability of network. Through the functions of centralized control plane, the open capabilities of network programming, SDN can reduce or even get rid of the limitations of the network infrastructure and architecture to improve the network efficiency.

In the architecture of SDN, routing control and topology control are still the core functions of the control plane. Centralized control of the routing causes some shortcomings, such as the bottlenecks of performance, a failure of single point, and poor scalability. We will research the deployment of routing protocols in distributed control plane of SDN in this paper. Currently, Kandoo is a hot spot of distributed architecture of SDN; we will research the crucial problem of routing protocol based on distributed architecture of Kandoo, such as the internal communication in distributed control plane and the deployment of distributed routing protocol.

The paper is organized as follows. Firstly, we research the distributed architecture of Kandoo which changes the traditional architecture of single control unit to form a distributed control plane architecture of multicontrol unit, thereby to achieve interconnection of multiple controller units. Then, we will analyze the distributed routing protocol of SDN and research the efficient implementation and deployment of distributed control plane. Then, we improved and optimized the two levels of controller of Kandoo based on the idea of RCP and analyzed the BGP and OSPF routing protocol using the embodiment of SDN distributed control plane. Finally, we give the simulation results, which show that our deployment strategies are superior to the traditional routing strategies.

## 2. Related Work

Forwarding and Control Element Separation (ForCES) Working Group (WG) in IETF Routing Area is one of the most influential research organizations in open programmable network research area [1]. The WG specializes in the architecture and protocol standards of open programmable IP network element (NE, such as router, firewall [2], or load balancer).

Open programmable networks are considered as the most prospective architectural approach to meet the above demands. In open programmable networks, a NE (e.g., a router/switch) is systematically separated into a control plane and a forwarding plane. Forwarding plane receives packets from outer networks, processes the packets according to functional requirements of the NE, and then outputs the packets back to outer networks. Forwarding plane usually needs the ability to process packets at line speed. Control plane controls forwarding plane for the whole forwarding process and provides adequate parameters for the process. More importantly, the interface between the control plane and the forwarding plane is standardized. Moreover, resources at the forwarding plane, which are used to process packets, are also described in a standardized way [3]. As a result, control plane can access and control the forwarding plane resources in a standard way. This makes it feasible for control plane and forwarding plane to be separated at their product level; that is, control plane and forwarding plane as separate products from different vendors can work together to form one NE with full interoperability [4]. On this basis, ForCES achieves the separation of the control software of the device and the underlying hardware physically and the virtualization of a variety of basic network functions module. The ForCES Working Group has completed the formulation of the ForCES Requirements, the ForCES Framework. The formulation of ForCES protocol and ForCES FE Model has been basically completed [5].

Before the concept of SDN is proposed, ForCES has already become the crucial technology of Forwarding and Control Element Separation, and, on the basis of ForCES, SDN will get more adequate theoretical support.

## 3. SDN Distributed Control Plane Architecture

**Figure 1:**
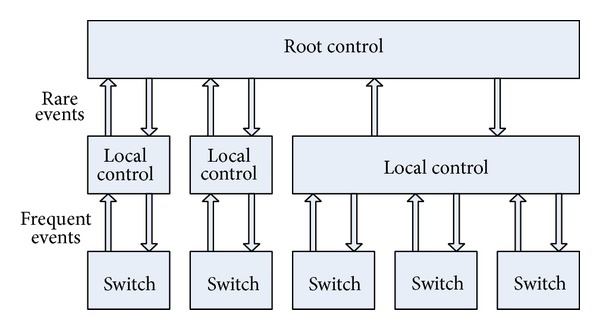
Kandoo two levels of controllers [4].

Architecture of Kandoo has changed the traditional structure of a single control unit and formed a distributed multicontrol plane architecture by interconnecting to multiple controller units [6]. Control plane of Kandoo can distinguish local controller applications from nonlocal applications substantially. Kandoo establishes a two-level controller: (a) local controller which performs local application as close as possible to the switch and (b) running logic centralized root controller of nonlocal application. As shown in Figure 1, on the one hand, a number of local controllers are deployed throughout the network, and each controller controls one or a small number of switches. On the other hand, the root controller controls all local controllers.

## 4. Distributed Routing Protocol for SDN Architectures

**Figure 2:**
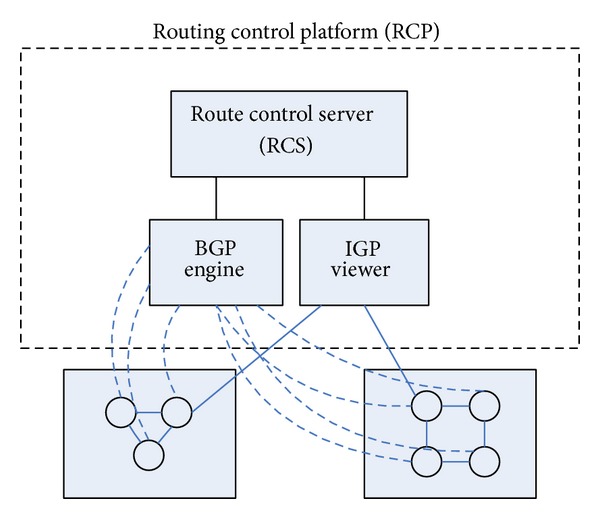
Schematic structure of the routing control platform.

Before the advent of Kandoo architecture, MIT and AT & T have proposed the idea of separating routing from router [7]. According to these thoughts, they proposed the routing control platform (RCP). This architecture is based on the circumstances in which the network topology and the corresponding management strategies deal with routing and exchange the reachable message between different autonomous domains. As shown in Figure 2, the control platform consists of three modules: IGP indicator, BGP routing engine, and router control server. Network distributes the functions of measurement and management through a number of different routers. But it is difficult to quickly perform the strategy of wide area network or deploy new services. As for such questions, RCP provides the direct capabilities of network control for the network operator, rather than indirectly affecting the network through a router. RCP can reduce the router's configuration status remarkably, thereby decreases the configuration errors and diminishes the complexity of network software management, and can deploy application services quickly.

**Figure 3:**
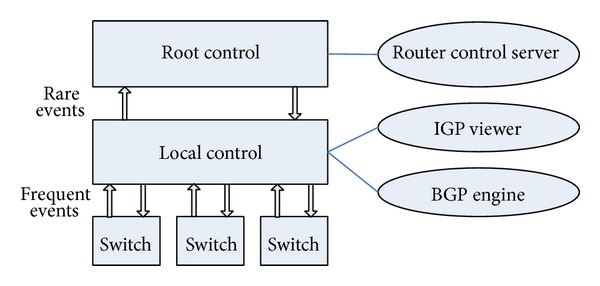
Improved Kandoo architecture based on RCP.

Inspired by the thought of the controller function modules of the RCP architecture, we improved and optimized the two levels of controllers of Kandoo and analyzed the more efficient implementation of the routing process of BGP and OSPF protocols in the distributed control plane of AS autonomous system; the specific structures are shown in Figure 3.

In the following, we will describe the implementation details of local controller and the root controller on distributed BGP protocol.

### 4.1. The Function Expansion of Local Controller

The local controller completes the function of IGP indicator and BGP engine.

The IGP indicator can monitor the IGP topology and provide the information to the root controller; the IGP indicator also can create IGP adjacency to accept the link state advertisements (LSAs) of IGP. In order to ensure the IGP indicator does not route packets, we set a big IGP weight between the IGP indicator and the routers. The IGP indicator can keep the newest topology state of IGP.

The BGP engine can maintain the iBGP sessions of each router in the AS system. These iBGP sessions allow the root controller to understand the candidates of routing. The BGP engine also can communicate the routing decisions with other routers. The iBGP runs over TCP, so the BGP engine does not need to be adjacent to each router physically.

We make a reasonable assumption as the connecting of the two IGP endpoints is sufficient to establish a BGP session. In fact, the continuing obstruction and incorrect configuration will affect that assumption, but these situations are unusual cases. Usually, the router will configure the BGP packets to forwarding path with high priority in order to ensure the transmission of these packets.

In order to accept the BGP updating, the root controller will send the BGP routing to the router using iBGP session. Because BGP updating has the property of next hop, the BGP engine can advertise BGP routing to other routers with the next hop. This feature does not allow BGP engine to forward packets. The BGP routing usually carries the attribute of next hop based on the egress router. Therefore, the root controller can send the routing to the router, the router's next hop is not changed, and the router can forward the packets to the exit routing.

The interaction of BGP engine and a router is the same as the interaction of the BGP spokesman and the router. But the BGP engine can send a different routing to a router. After choosing a new optimal routing from the neighbor AS, a router will send the BGP updating to the BGP engine. Similarly, BGP engine sends the update message only when the routing of a router needs to change.

### 4.2. The Function Expansion of Root Controller

Root controller accepts the message of IGP topology and BGP routing from the local controller, then calculates the best routing for a group of routers, and assigns the results to the appropriate router through the BGP engine. According to the selection process of BGP routing, in the first step of the route table, the best routing has been selected from a number of candidates of routers. And the root controller no longer assigns the routing to the router. In order to make the right routing decisions for a group of routers in the same partition, it must meet the condition in which the root controller must be able to receive the topology message of IGP and the routing message of BGP in this partition.

Although the root controller has considerable excellent flexibility in assigning routes, a more reasonable approach is to choose routing in condition of iBGP configuration of the whole network. For the purpose of simulating an iBGP configuration of the whole network, according to Algorithm 1, the root controller needs to perform BGP routing process.

The reasons that root controller can perform calculations are as follows.

- It knows the IGP topology; the root controller can select the reachable egress routers from their visible routers in partition.
- From step 1 to 4, the corresponding property is compared through BGP message.
- Step 5: the root controller learns about message of iBGP through other routers and considers learning eBGP message.
- Step 6: the root controller compares the path cost of IGP via the message which IGP engine publishes.
- Step 7: because iBGP message maintains each router and the BGP engine, the root controller knows the routing ID of each router. By calculation, the root controller sends the proper routing to each router.

## 5. The Deployment Strategy of Distributed Routing Protocol of SDN

### 5.1. The Deployment of Distributed BGP on the Root Controller of SDN

**Figure 4:**
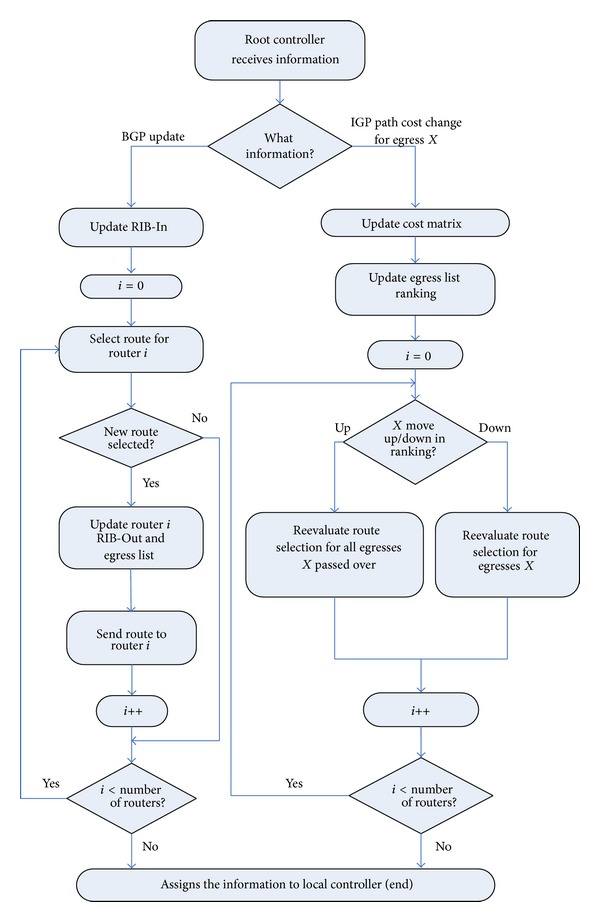
The implementation process of BGP protocol on Kandoo.

Root controller acts the functional roles as BGP speaker of AS. Root controller receives the information from the local controller.  Figure 4 shows the processes of the implementation of root controller. Root controller receives the updating message from the local controller, and the learned routing message is stored in the routing table. Root controller performs the routing of each router and stores the selected routing in the RIB-Out table. The RIB-In table maintains the routing cluster of each prefix, and each BGP has the property of next hop to uniquely identify the egress router. Root controller also accepts the cost of IGP path of each router from the local controller. Root controller calculates the optimum message of BGP routing using the RIB-In table, then assigns the information to local controller, and sends updating message to the router. When you receive the change of cost information of the path from the local controller, if the root controller makes the decisions of selecting the best routing using step 6, namely, considering the cost of IGP path, the root controller should recalculate the best routing.

To improve the ability of calculating the routing message of the root controller, the method is shown as follows.

**Figure 5:**
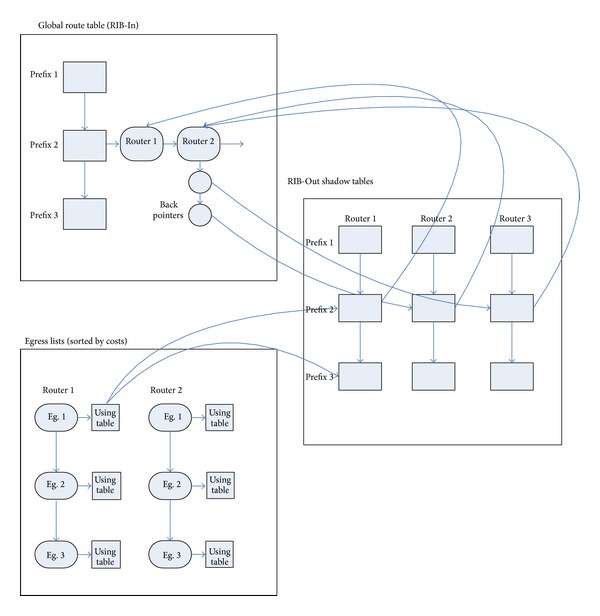
Data structures of RIB-Out and RIB-In route table and egress lists of root controller.

When root controller calculates the best routing, finding the affected routing is actually a process of high price. We present a method for efficient execution based on level arrangement of the egress router. The details of the method are shown in Figure 5.

#### 5.1.1. Only Storing One Copy of the BGP Routing

It needs a large number of additional stored overheads to store the detached copy of BGP routing of each destination prefix. In order to reduce the storage overhead, root controller routing message is stored only in RIB-In table. The property of next hop of BGP routing can uniquely identify the egress router. According to the updating packets, root controller searches the RIB-In table based on the prefix and adds, updates, or deletes the corresponding routing based on the property of next hop. To implement the RIB-Out table, root controller table should regard the router images containing RIB-In pointer as a search tree of prefix. Figure 5 shows two examples of how to implement the RIB-Out from the RIB-In pointer.

#### 5.1.2. Keeping in Touch with the Routers Which Have Been Assigned to Each Routing

When one routing path is withdrawn, root controller needs recalculation of a new routing for the router which is using the routing path. In order to identify the affected routers quickly, the route which is stored in the RIB-In table should contain a reverse pointer list pointing to the router. For example, in the RIB-In table of Figure 5, routing path 2 with the prefix 2 has reverse pointer indicating that router 2 and router 3 have been assigned to that path.

#### 5.1.3. Maintaining One Array of Egress Based on the Cost of IGP Path for Each Router

The change of single IGP path cost may affect the BGP decision of root controller for the egress routing of the destination address prefix. In order to avoid the reselection of routing for every prefix and every router, root controller needs to maintain a level arrangement of egress router, as shown in Figure 5, the egress lists. For each egress, root controller stores the pointer to the prefix and route link. For example, router 1 arrives at prefixes 2 and 3 through egress 1. If the IGP path cost increases from router 1 to egress 1, the BGP speaker puts down the level of egress 1, until it comes up with the higher one. Finally, the root controller recalculates the prefix of egress 1 to get the BGP routing.

#### 5.1.4. Assigning Routing to a Group of Associated Routers

Each router does not need to calculate the BGP routing, and the root controller can assign the BGP routing to a group of routers with the same destination prefixes.

### 5.2. The Deployment of Distributed OSPF on Local Controller

**Figure 6:**
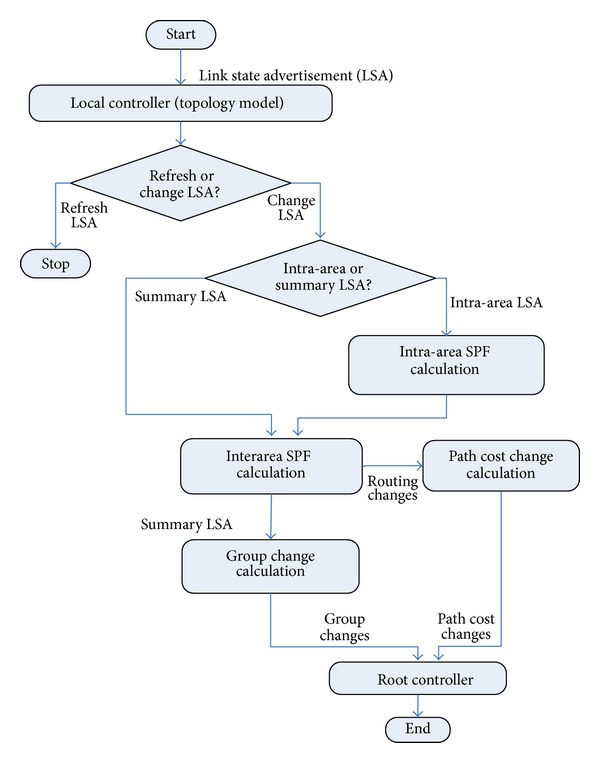
The implementation process of OSPF protocol on local controller.

Local controller connects one or more routers to accept the link state advertisement. While local controller accepts the message of BGP routing, it sends the BGP routing messages to the root controller and also sends BGP routing to single router. Local controller maintains a newest network topology and calculates the path cost of each pair of the routers.  Figure 6 shows the principle of the running condition of OSPF protocol on the local controller of SDN.

Through analyzing the architecture of Kandoo, we know that one of the important works of the local controller is to download the task of the root controller. We will study the methods of how to change local controller to reduce the load of root controller. To improve the ability of downloading the load of root controller to the local controller, the methods are studied as follows.

#### 5.2.1. Only Sending the Change Information of Path Cost

Even if the network is in a stable condition, local controller not only creates LSA according to the changes of network, but also regularly updates the LSAs message. Local controller maintains the steady status of network according to the topology model and determines whether to change or not the network topology of the updating LSA. For the changed LSA, local controller calculates the shortest path to determine the newest cost of path from the perspective of each router. Local controller does not send the information of all the paths to root controller and only sends part of the information which has been changed through the calculation of path cost.

For the purpose of scalability, OSPF domain is divided into several regions to form the topology with a radiation center. Area 0, as the backbone of the region in the center, provides connections to other nonbackbone areas to form radiation. Each link belongs to a determined area. A router connecting multiple regions is known as the backbone router, which is also known as local controller. The local controller will learn the whole topology of the area. The local controller does not learn the topology of other remote areas; but it will learn all the information of path cost to a remote routing node.

Local controller performs the shortest path first (SPF) algorithm according to the whole topology; it looks like ignoring of the domain boundary, but OSPF will assign the path of the routers belonging to the domain to be inside the domain, despite the existence of the shortest path of cross domain boundaries. So, it does not ignore the problem of domain boundary in the process of calculation, therefore using two stages to calculate. The first stage, called the inside-domain phase, as shown in Figure 6, calculates the path cost for each domain using the LSAs message within the domain. In the second stage, called outside-domain stage, local controller calculates the path cost for the router in different domains through the integration of paths.

#### 5.2.2. Reducing the Load of Root Controller through Aggregation Router

Local controller can use the structure of domain to reduce the number of routers of root controller. To achieve this purpose, local controller requires (1) provision of the path cost information of nonbackbone domain routers and backbone domain routers and (2) the formation of each of the nonzero domains as a router group and its provision of information of the group. Furthermore, local controller need not be physically connected to a nonzero domain, because the total LSAs message obtained from the backbone domain allows the local controller to calculate the path cost from the router of the zero domain to the other routers. Meanwhile, local controller will determine the group relationship of routers from the total message of LSAs.

#### 5.2.3. Caching BGP Routing Message

Local controller stores the message of RIB-In and RIB-Out table locally. When a failure of BGP speaker occurs, the caching of RIB-In will bring in a new duplication or recover rapidly according to the latest duplication in the condition of no influence to the routers. When the connection of IGP is interrupted temporarily, the caching of RIB-Out will resend the routing to this router.

#### 5.2.4. The Low-Level Exchanges of Managing the Router

Local controller creates simple and stable communication with other routers through a number of flow tables to maintain the BGP sessions and multiplexes the updating message to form a single flow to send to the root controller.

## 6. Simulation

**Figure 7:**
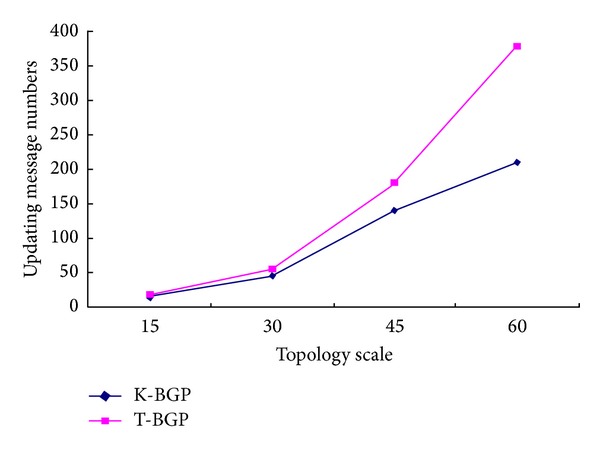
Comparison of numbers of updating message based on difference topology scale.

**Figure 8:**
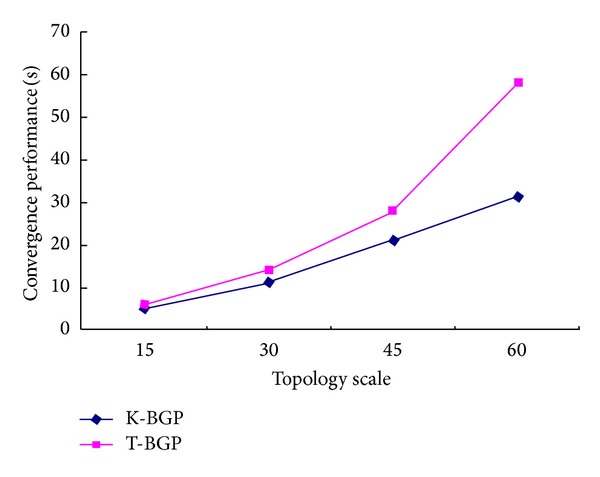
Convergence performance comparison based on difference topology scale.

In order to evaluate the performance of BGP under the Kandoo architecture, we simulate the Kandoo topology using Cisco GNS3. We, respectively, simulate 5 root controllers (or AS), 30 root controllers, 45 root controllers, and 60 root controllers based on the Kandoo architecture. And each root controller connects 6 local controllers. We compare the convergence performance and the numbers of updating message of the Kandoo-BGP (K-BGP) with the traditional BGP (T-BGP). The results are shown in Figures 7 and 8.

From the simulation results of Figure 7, we can see that the numbers of updating message of Kandoo-BGP are less than traditional BGP based on difference topology scale; in particular, when the topology scale is larger and larger, the numbers of updating message significantly decrease.

From the simulation results of Figure 8, we can see that the convergence performance of Kandoo-BGP is superior to traditional BGP based on difference topology scale; in particular, when the topology scale becomes larger, the convergence performance of K-BGP significantly increases.

## 7. Summary

This paper mainly researches the deployment of routing protocols in distributed control plane under SDN. The distributed characteristics of Kandoo are studied deeply, which is achieved by multiple controller units interconnected to form a distributed control plane architecture of multicontrol unit. We improved and optimized Kandoo's two levels of controllers based on ideological inspiration of RCP and analyzed the implementation and deployment of BGP and OSPF protocol in a distributed control plane of SDN. We give the simulation results, which show that our deployment strategies are superior to the traditional routing strategies.

Although the deployment strategies have achieved the desired goal, because of the constraints of time and the objective conditions, there are still some deficiencies in this paper. The future works carried out are (1) continuing to deepen the study of a variety of control architectures of SDN and extending the function of Kandoo so that it can deploy and implement efficiently and (2) researching the interdomain deployment strategy of BGP protocol combined with the distributed control plane architecture of SDN and making BGP to fully implement distributed characteristics.

## Figures and Tables

**Algorithm 1:**
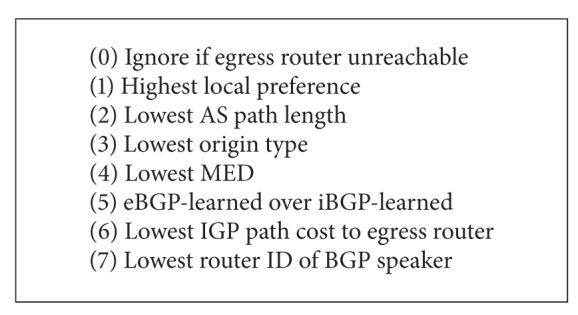
Steps of the process of BGP route selection.
